# Interplay between YB-1 and IL-6 promotes the metastatic phenotype in breast cancer cells

**DOI:** 10.18632/oncotarget.5664

**Published:** 2015-10-15

**Authors:** Bàrbara Castellana, Trond Aasen, Gema Moreno-Bueno, Sandra E. Dunn, Santiago Ramón y Cajal

**Affiliations:** ^1^ Molecular Pathology, Vall d'Hebron Research Institute (VHIR), Universidad Autonoma of Barcelona, Barcelona, Spain; ^2^ Departments of Department of Obstetrics and Gynecology, Child and Family Research Institute, University of British Columbia, Vancouver, BC, Canada; ^3^ Instituto de Investigaciones Biomédicas “Alberto Sols” CSIC-UAM, Madrid, Spain; ^4^ Phoenix Molecular Diagnostics Ltd., Richmond, BC, Canada

**Keywords:** Y-box binding protein 1, interleukin-6, breast cancer, invasion, migration

## Abstract

Epithelial to mesenchymal transition (EMT) induces cell plasticity and promotes metastasis. The multifunctional oncoprotein Y-box binding protein-1 (YB-1) and the pleiotropic cytokine interleukin 6 (IL-6) have both been implicated in tumor cell metastasis and EMT, but via distinct pathways. Here, we show that direct interplay between YB-1 and IL-6 regulates breast cancer metastasis. Overexpression of YB-1 in breast cancer cell lines induced IL-6 production while stimulation with IL-6 increased YB-1 expression and YB-1 phosphorylation. Either approach was sufficient to induce EMT features, including increased cell migration and invasion. Silencing of YB-1 partially reverted the EMT and blocked the effect of IL-6 while inhibition of IL-6 signaling blocked the phenotype induced by YB-1 overexpression, demonstrating a clear YB-1/IL-6 interdependence. Our findings describe a novel signaling network in which YB-1 regulates IL-6, and *vice versa*, creating a positive feed-forward loop driving EMT-like metastatic features during breast cancer progression. Identification of signaling partners or pathways underlying this co-dependence may uncover novel therapeutic opportunities.

## INTRODUCTION

Despite major advances in the past 40 years, the inability to cure metastatic disease remains a significant hurdle to effective cancer therapy. It is also usually the underlying cause of breast cancer death. Metastasis is a complex process in which metastatic cancer cells disseminate from primary sites and invade secondary target organs such as the lungs, bone, and brain [1]. One step toward tumor progression is the epithelial to mesenchymal transition (EMT) [2–4]. The EMT, orchestrated by the upregulation of oncogenic transcription factors such as TWIST1/2, SNAI1/2, and ZEB1/2 facilitates the acquisition of mesenchymal characteristics, such as plasticity, migratory capacity, and invasiveness, and allows cancer cells to escape from the primary tumor to form distant metastasis.

Y-box binding protein 1 (YB-1) is a highly regulated protein with multiple localization-dependent functions. In the nucleus, YB-1 acts as a transcription/splicing factor, whereas, in the cytoplasm, it binds to and stabilizes mRNA in order to regulate translation [5]. In the last decade, YB-1 has been identified as a principal factor in many malignant tumors [6, 7] and is involved in many of the “hallmarks of cancer” proposed by Hanahan and Weinberg [8]. Elevated YB-1 protein levels have been associated with poor prognosis and drug resistance [9], relapse [10], progression [11], induction of cancer stem cell-like features [12], and metastasis [13]. YB-1 is overexpressed in breast cancer, especially in the aggressive triple-negative (TNBC) subtypes, almost 70% of which are strongly positive for YB-1 [10]. Davies and coworkers [14] elegantly showed that YB-1 contributes to the conversion of hormone receptor-positive breast cancer cells to the TNBC phenotype.

During tumor progression, the tumor microenvironment favors the release of proinflammatory cytokines. The combination of intrinsic tumor characteristics and diverse environmental cues can contribute to the metastatic process. Abnormal cytokine signaling plays a key role in cancer metastasis by creating the appropriate inflammatory microenvironment [15]. Of the cytokines participating in the cancer process, interleukin 6 (IL-6) has been shown to promote breast cancer metastasis [16–18], and high IL-6 levels are associated with poor clinical outcomes in breast tumors [19]. Activation of IL-6 triggers three signaling pathways: (i) JAK/STAT3, (ii) PI3K/AKT/mTOR, and (iii) Ras Raf/MEK-ERK/MNK. These pathways converge in autocrine or paracrine phosphorylation of STAT3, making IL-6 a key contributor to tumor growth, drug resistance, induction of cancer stem cells, and metastasis [17]. Additionally, IL-6 itself [20, 21] and components of its signaling pathway, including JAK/STAT3 [22], MEK1 [23], and PI3K/AKT [24], can induce EMT. Notably, expression of estrogen receptor (ER) correlates inversely with that of IL-6 [25], meaning that ER-negative breast cancers, including TNBC subtypes, produce more IL-6 than ER-positive tumors [26, 27] and are able to exploit both paracrine and autocrine IL-6 signaling. Similarly, TNBCs express high levels of active YB-1 [9, 28] and IL-6, whereas ER-positive cell lines have lower levels of both proteins.

Recently, YB-1 has been shown to promote metastatic features such as migration, invasion, and EMT characteristics [29–33]. However, its interplay with other signaling pathways regulating EMT processes, such as cytokines, remains poorly studied.

In the present work, we explored the relationship between YB-1 and IL-6 signaling in the acquisition of EMT characteristics by breast cancer cells. Our results show that YB-1 increases cell migration and invasion, in a process that appears to require the concomitant induction of IL-6 production. Moreover, induction of EMT by IL-6 also requires the upregulation of YB-1. Our results describe a novel mechanism in which YB-1 induces metastatic characteristics through IL-6 expression, and vice versa.

## RESULTS

### YB-1 overexpression promotes cellular migration and invasion in breast cancer cells

**Figure 1:**
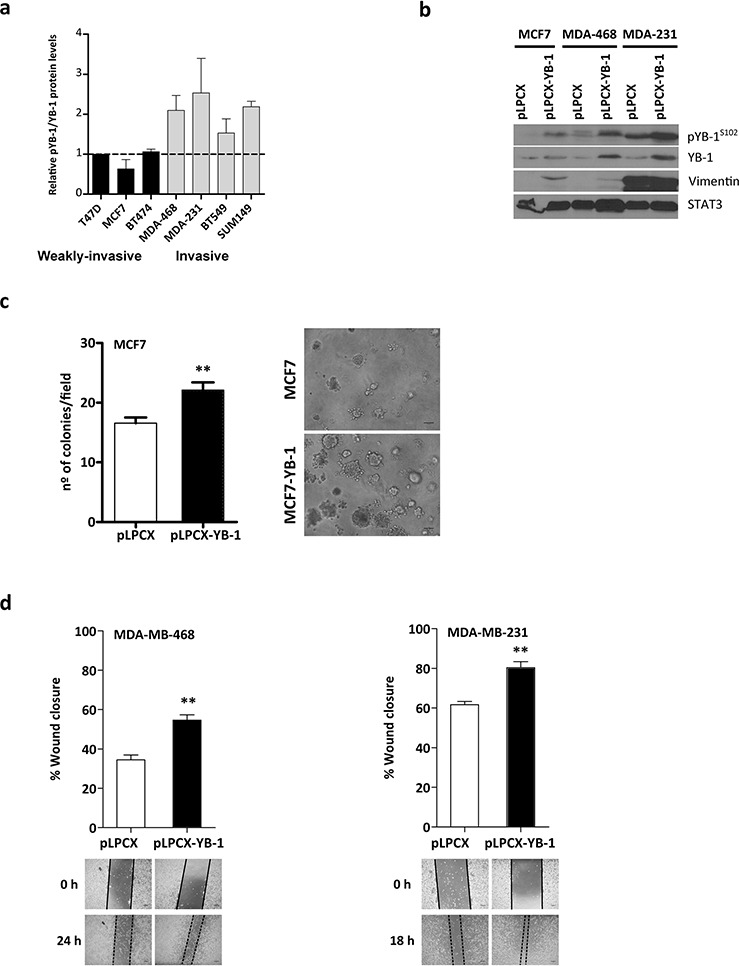

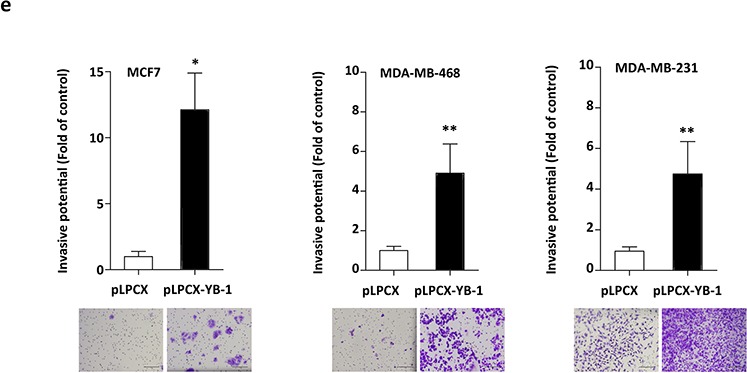
YB-1 promotes migration and invasion in breast cancer cells **a.** Western blot analysis of breast cancer cells lines showing their phosphoYB-1 (pYB-1^S102^) and total YB-1 protein levels. **b.** Effects of forced expression of YB-1 in breast cancer cells. Cells were stably transfected with plasmids (5 μg) expressing YB-1 (pLPCX-YB-1) or an empty control plasmid (pLPCX), as indicated. The levels of the mesenchymal marker vimentin, pYB1^S102^, and total YB-1 are shown. **c.** Semi-fluid basement membrane assay of MCF7 and MCF7^YB-1^ cells. Colonies formed after incubation for 10 days. *P < 0.05 versus control by Student's *t* test. Representative photomicrographs of colonies formed by the cells growing in Matrigel. Scale bar, 160 μm. **d.** Cell migration was studied with a scratch-wound assay in MDA-MB-468 and MDA-MB-231 cells expressing control and YB-1 vectors (mean ± SEM; **P < 0.001 by Tukey's post-hoc test. **e.** Matrigel invasion assay in MCF7, MDA-MB-468, and MDA-MB-231 cells stably expressing the two different plasmids described above. Cells were harvested after 16 h and 5 × 10^4^ cells were seeded onto the Transwell invasion chamber for 24 h. Cells that crossed the Matrigel-coated filter were fixed, stained, and counted. Representative bright-field images of the bottom surface are shown. Six random microscopic fields were counted for each group. Results are presented as the fold change over each parental cell line (mean ± SEM; *P < 0.05, **P < 0.01 by Student's *t* test).

Aggressive and metastatic breast tumors, such as TNBC, express high levels of YB-1 [34]. To determine whether YB-1 levels correlate with an invasive phenotype, we compared highly invasive breast cancer cell lines with those that were weakly invasive or non-invasive (Figure 1A). Invasive breast cancer cell lines (MDA-MB-231, MDA-MB-468) expressed higher levels of pYB-1^S102^/total YB-1 than non-invasive cell lines (T47D, MCF7) (Figure 1A).

Next, we sought to determine the role of YB-1 in the acquisition of metastatic features. We stably infected three breast cancer cell lines (MCF7, MDA-MB-468, and MDA-MB-231) with retrovirus expressing YB-1 (pLPCX-YB-1) or with control empty vector (pLPCX). We verified forced expression of YB-1 at both RNA (Figure S1A) and protein (Figure 1B) levels. Forced expression of YB-1 in MCF7 cells (MCF7^YB-1^) increased the number of cell colonies compared with control cells (*P* = 0.004; Figure 1C). Additionally, YB-1 overexpression induced EMT features, including transcriptional upregulation of *SNAI1* mRNA (Snail1, an E-cadherin repressor) and downregulation of *CDH1* mRNA (E-cadherin) (Figure S1B).

Because cell migration and invasion are important characteristics acquired during the EMT process [35], we performed migration wound-healing experiments in serum-free medium to exclude interference from the proliferative action of YB-1. Consistent with the changes in gene expression, MDA-MB-468^YB-1^ and MDA-MB-231^YB-1^ cells showed increased cell motility (*P* = 0.001 and *P* = 0.008; Figure 1D). MCF7 cells did not display any appreciable migration for the duration of this assay and were therefore excluded (data not shown). Consistent with a role for YB-1 in promoting EMT-like features, MDA-MB-468^YB-1^ and MDA-MB-231^YB-1^ cells displayed ~4.5-fold increased invasion rates compared with control cells (*P* = 0.001; Figure 1E). In MCF7 cells, increased YB-1 expression levels significantly activated cell invasion (12-fold, *P* = 0.025). These results indicate that YB-1 increased the motility and invasive capacity of breast cancer cells, implying a role for YB-1 in breast cancer progression and metastasis.

### Loss of YB-1 promotes an epithelial-like phenotype

**Figure 2:**
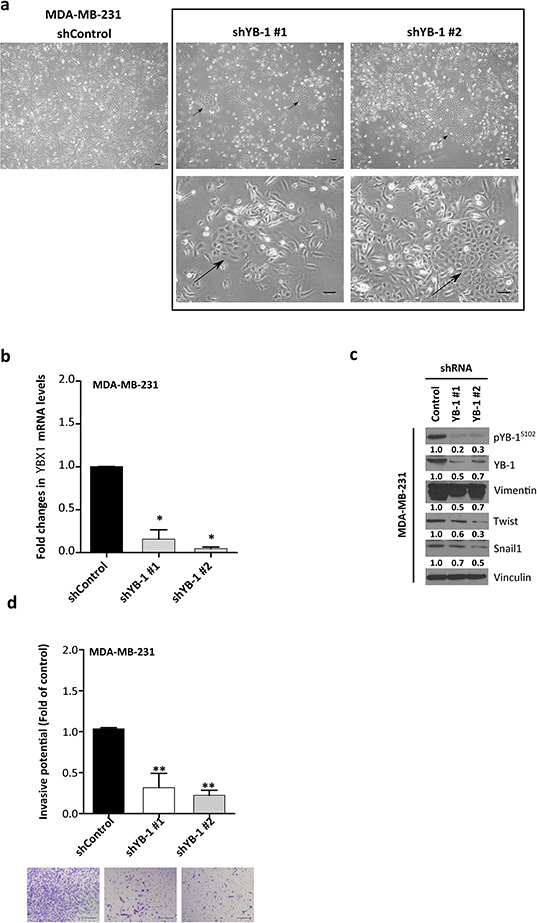
YB-1 knockdown reverses EMT features toward a more epithelial-like phenotype in MDA-MB-231 cells **a.** Morphologic changes in MDA-MB-231 cells after infection with shRNA-YB-1#1 and #2 or empty vector. Arrows indicate clusters of cells with epithelial-like morphology. **b.** qRT-PCR determination of *YBX1* mRNA. The transcript levels of cells transfected with control shRNA were set as 1. Means ± SEM are shown for three independent experiments. *P < 0.05, **P < 0.01 compared with cells transfected with control shRNA by Tukey's test. **c.** Western blot analysis was performed in shRNA-infected MDA-MB-231 cells. **d.** Transwell invasion assay showed a diminished invasive capacity after YB-1 knockdown. Data are presented as mean ± SEM. *P < 0.05, **P < 0.01 by Tukey's test.

Given the proposed functional involvement of YB-1 in the EMT [30, 32, 33], we asked whether YB-1 knockdown would decrease the mesenchymal characteristics of MDA-MB-231 cells. This cell line has undergone EMT and has considerable levels of total and pYB-1^S102^ (Figure 1A). We infected MDA-MB-231 cells with lentiviral vectors (short hairpin RNA [shRNA] control or two shRNAs targeting YB-1). After 4 days of antibiotic resistance selection, cells formed clusters with a more cuboidal epithelial-like morphology (Figure 2A). MDA-MB-231 cells transfected twice with YB-1 two different short interfering RNAs (siRNAs, targeting YB-1) showed similar morphological changes (Figure S2A and S2B). Quantitative real-time PCR (qRT-PCR) and Western blot confirmed the inhibition of YB-1 (Figure 2B and 2C). These changes in cellular appearance were mirrored by the concurrently decreased levels of the mesenchymal marker vimentin and the EMT inducers Snail1 and Twist (Figure 2C) and reduced invasiveness (*P* = 0.001; Figure 2D). In summary, YB-1 knockdown is sufficient to reverse the phenotype of cancer cells and make them regress and adopt a more epithelial phenotype.

### IL-6 induces YB-1 expression and activation

IL-6 induces and maintains the EMT through feed-forward loops in a variety of carcinomas, including breast [20, 36]. The main biological effects of IL-6 are mediated by activation of the STAT3 pathway. STAT3 needs to be phosphorylated at tyrosine residue 705 (pSTAT3^Y705^) to allow the formation of dimers that translocate to the nucleus to regulate gene transcription [37]. Many cancer cell lines show nuclear STAT3 translocation with subsequent transcriptional activation of programs such as the EMT. We analyzed a large number of breast cancer cell lines with Gene Expression-Based Outcome for Breast Cancer Online (GOBO) [38], finding that TNBC and HER2-positive breast cancer cell lines express higher levels of *YBX1* and *STAT3* (Figure S3A). TNBC cell lines had abundant levels of pSTAT3^Y705^ protein based on immunoblotting (Figure S3B).

**Figure 3:**
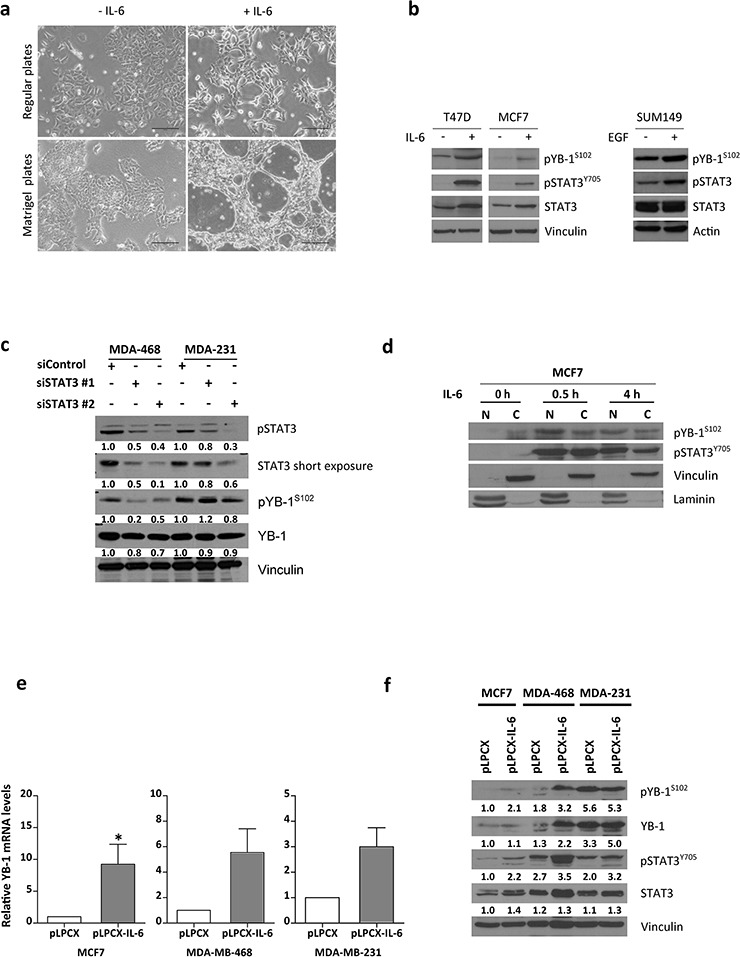
IL-6 induces YB-1 phosphorylation in MCF7 cells **a.** Phase contrast photomicrographs of cells treated with IL-6 (50 ng/mL, 10 days) cultured in regular plates (top) or collagen type I-coated plates (below). MCF7 cells undergoing EMT-like morphologic changes. Scale bar, 161 μm. **b.** Western blot assessing pYB-1^S102^ expression under IL-6 stimulus in breast cancer cell lines. Phospho-STAT3 (pSTAT3^Y705^) indicates STAT3 activation in the IL-6 signaling pathway. **c.** Western blot of MDA-MB-468 and MDA-MB-231 cells transfected for 96 h with STAT3 siRNAs. Levels of pSTAT3, pYB-1^S102^, and total YB-1 are shown. **d.** Subcellular localization of pYB-1^S102^ after IL-6 (25 ng/mL) treatment in MCF7 cells. N, nuclear, C, cytoplasm. **e.** *YBX1* mRNA levels assessed by qRT-PCR in IL-6–overexpressing cells. **f.** Western blot analysis of breast cancer cells overexpressing IL-6.

To study the effect of IL-6 on YB-1, we stimulated MCF7 cells with recombinant human IL-6 (25 ng/mL) to induce an EMT-like phenotype [20]. MCF7 cells had low levels of pYB-1^S102^ (see Figure 1A) and respond to IL-6 via the IL-6 receptor components alpha (gp80) and gp130 [27]. Control MCF7 cells displayed a characteristic cobblestone and epithelial morphology, whereas cells treated with IL-6 for 10 days displayed a more fibroblastic morphology with scattered distribution, suggesting a partial activation of the EMT program (Figure 3A). In accordance with these changes, IL-6–stimulated cells displayed increased *YBX1* and *SNAI1* mRNA levels and decreased *CDH1* mRNA levels (Figure S3C). IL-6 treatment also increased pYB-1^S102^ levels as well as activated STAT3 (Figure 3B) in a similar fashion to epidermal growth factor (EGF) treatment (Figure 3B). Upregulation of pYB1^S102^ by IL-6 was in a dose-dependent manner (Figure S3D).

The specificity of YB-1 phosphorylation by IL-6 was checked by siRNA-mediated knockdown of STAT3 in MDA-MB-468 cells. This cell line produces IL-6 and can respond to autocrine IL-6 signaling. Cells with silenced STAT3 had reduced pYB-1^S102^ levels (Figure 3C). Subsequent YB-1 activation partially required STAT3 phosphorylation by IL-6. Together, this suggests that YB-1 is activated (pYB-1^S102^) by the STAT3 pathway (via various upstream pathways), which may represent a common feature among breast cancer cells.

Phosphorylation of YB-1 can lead to nuclear translocation in cancer cells [39]. In basal conditions, pYB-1^S102^ localized mainly to the cytoplasm. After IL-6 treatment, pYB-1^S102^ translocated to the nucleus within 30 min, maintaining its localization until at least 4 h. However, some pYB-1^S102^ remained in the cytoplasm of MCF7 cells (Figure 3D). YB-1 phosphorylation by IL-6 leads to changes in intracellular localization. On the other hand, forced IL-6 expression increased *YBX1* mRNA (Figure 3E) and total YB-1/pYB-1^S102^ protein (Figure 3F) levels.

### Induction of EMT-like characteristics requires YB-1

**Figure 4:**
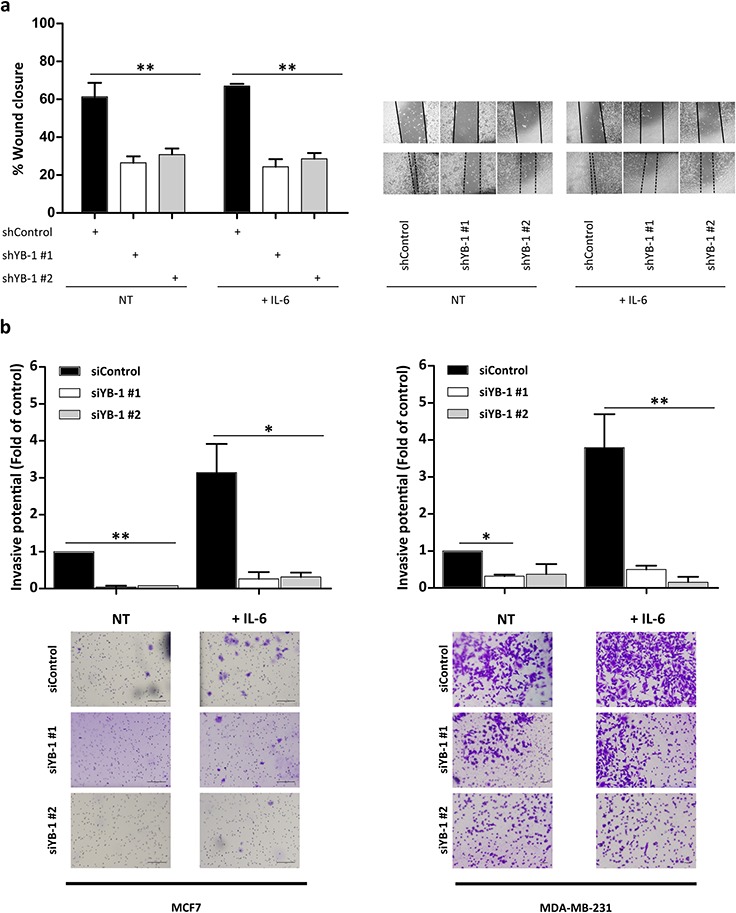

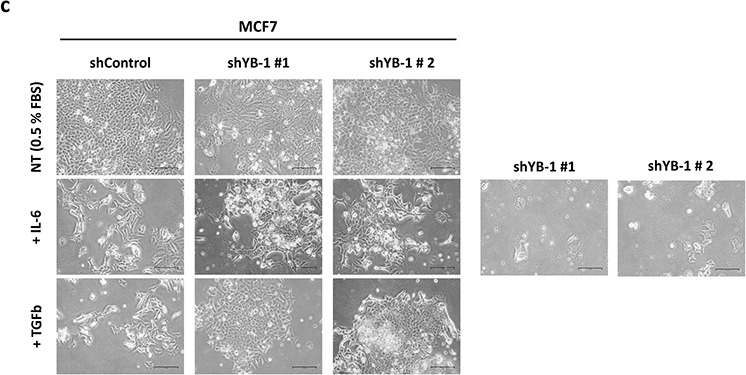
YB-1 is involved in the IL-6–induced metastatic phenotype **a.** Wound healing migration assays in shControl (pLKO-shControl)- and shYB-1 (pLKO-shYB-1)-infected MDA-MB-231 cells (left) without or with IL-6 treatment. Representative photographs of three independent experiments (right). **b.** Matrigel invasion assays of MCF7 and MDA-MB-231 cells transfected with 30 nM of siRNA control or two different siRNAs targeting YB-1. After 72 h, cells were seeded in Matrigel-coated Transwell invasion plates in the absence or presence of IL-6 for 24 h. Shown are representative pictures of three independent experiments (mean ± SEM; *P < 0.05, **P < 0.01 by Tukey's HSD). **c.** Phase contrast images show morphological changes in MCF7 cells expressing shControl or shYB-1 with or without IL-6 or TGFβ treatment for 13 days in collagen-coated plates. NT, non-treated.

To gain insight into the relationship between YB-1–and IL-6–induced EMT-like features, we investigated the impact of YB-1 depletion on cellular migration and invasion in MDA-MB-231 cells with or without the presence of IL-6. Cells transfected with either siRNAs targeting YB-1 (short-term silencing; 96 h; Figure S4A) or with YB-1 shRNAs (long-term silencing; Figure 4A) had significantly lower migratory (Figure 4A and S4A; **P < 0.001) and invasive (Figure 4B; *P < 0.05, **P < 0.01) rates than control cells. Depletion of YB-1 blocked the increased migratory and invasive capacities caused by IL-6 stimulation (Figure 4B; *P < 0.05, **P < 0.01), suggesting that IL-6–induced migratory and invasive features require YB-1 signaling. Moreover, IL-6–stimulated cell lines displayed increased invasiveness (Figure S3E), suggesting a relationship between increased cell invasion and increased YB-1 expression.

Next, we silenced YB-1 in MCF7 cells treated with IL-6 or with TGFβ (a well-known regulator of EMT). After 13 days of treatment, control cells displayed EMT-like features, whereas YB-1–knockdown cells failed to undergo EMT-associated morphological changes (Figure 4C). Moreover, we observed increased cell death upon IL-6 stimulation in cells where YB-1 had previously been silenced. This suggests that YB-1 may be a critical mediator of EMT-like features induced by a variety of upstream pathways, including IL-6 and TGFβ.

### Forced expression of YB-1 increases IL-6 levels

**Figure 5:**
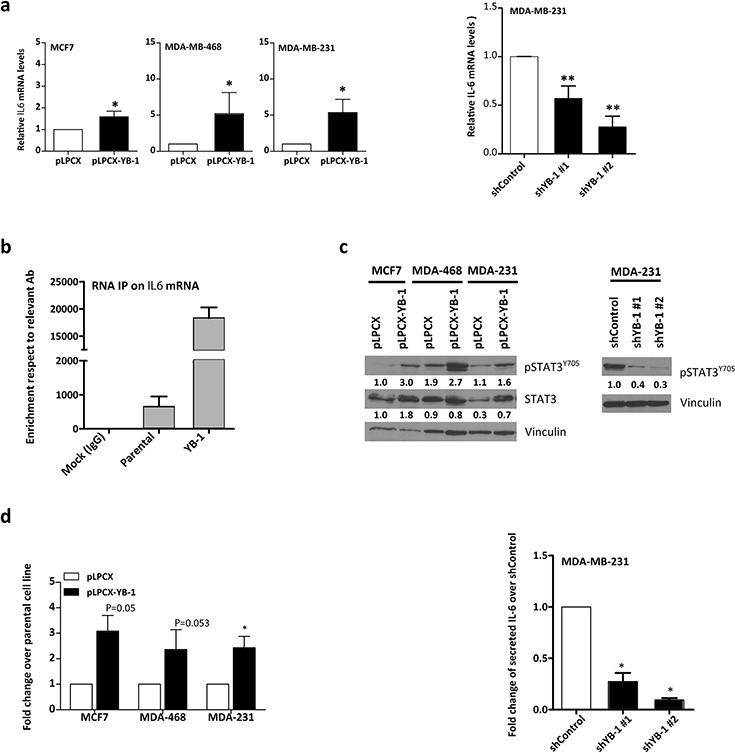
YB-1 drives IL-6 expression in breast cancer cell lines **a.** qRT-PCR analysis of *IL6* mRNA levels after forced YB-1 expression in MCF-7, MDA-MB-468, and MDA-MB-231 cells and in YB-1–depleted MDA-MB-231 cells compared with control cells. The indicated means are significantly different. *P < 0.05, **P < 0.01 by Tukey's test. **b.** RIP in MDA-MB-231^YB-1^ cells compared with control cells. **c.** On the left, Western blot analysis of cells transfected with YB-1 vector or control empty vector and, on the right, MDA-MB-231 cells transfected with shRNA of YB-1. **d.** IL-6 ELISA analysis of serum-free culture supernatants.

We wanted to determine whether YB-1 could activate IL-6 expression in breast cancer cells. In all three cell lines, changes in YB-1 levels increased *IL6* mRNA levels (Figure 5A; *P < 0.05, **P < 0.01). Cytokine expression is usually transient and *IL6* mRNA has a particularly unstable nature due to the presence of Au-rich elements (AREs) in its 3′UTR [40]. YB-1 has been described as a structural component of messenger ribonucleoprotein particles (mRNPs) [41, 42] that can act as an ARE RNA-binding protein, meaning that it can regulate the stability and/or translation of target mRNAs. We performed an RNA-binding protein immunoprecipitation (RIP) assay to analyze whether YB-1 could bind and stabilize *IL6* mRNA. As a control, we used *SNAI1* because YB-1 is known to bind and stabilize its mRNA (Figure S5A). We observed a striking increase in total *IL6* RNA levels bound to YB-1 protein in MDA-MB-231^YB-1^ cells (Figure 5B), suggesting that YB-1 may induce *IL6* mRNA stability via direct binding.

Overexpression of YB-1 increased pSTAT3^Y705^ in the cell lines tested (Figure 5C). Levels of pSTAT3^Y705^ can be indicative of IL-6 presence in the supernatant. To confirm this result, serum-free supernatants were tested by IL-6 ELISA. We confirmed that cells with forced YB-1 expression displayed 2–3-fold increased IL-6 levels in the supernatant (Figure 5D). In contrast, YB-1 depletion reduced IL-6 levels in the supernatant (Figure 5D and S5B).

These results indicate that YB-1 and IL-6 interplay at another novel level, so that YB-1 is not only regulated by IL-6, but IL-6 is also reciprocally regulated by YB-1. Thus, one mechanism used by YB-1 to induce mesenchymal characteristics could be modulation of IL-6 levels in the medium.

### IL-6 blockage abrogates YB-1 activation, cell migration, and invasion

**Figure 6:**
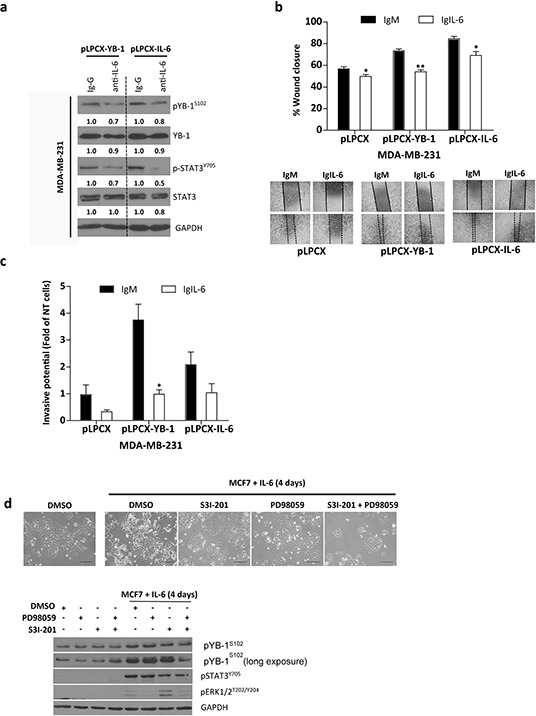
Involvement of IL-6 in YB-1–promoted migration and invasion Cells were incubated with 2 μg/mL anti-mouse as a negative control or 2 μg/mL anti–IL-6 antibody. **a.** Western blot analysis, **b.** cell migration, and **c.** cell invasion assays of control and MDA-MB-231^YB-1^ cells pretreated with anti-mouse or anti–IL-6 antibody for 20 h. Values are the mean ± SEM of the fold change over MDA-MB-231 non-treated cells. *P < 0.05, **P < 0.001 by Tukey's tests. **d.** Treatment of MCF7 cells with inhibitors of MEK (50 μM PD98059) and/or STAT-3 (100 μM S3I-201) significantly inhibited IL-6–induced EMT-like phenotype. Phase contrast photomicrographs. Scale bar, 161 μm. NT, non-treated.

To determine the contribution of IL-6 to YB-1–induced migration and invasion, we blocked IL-6 signaling. The presence of an IL-6 monoclonal antibody that neutralized secreted IL-6 reduced the levels of both pYB-1^S102^ and pSTAT3^Y705^ (Figure 6A). Anti–IL-6 treatment blocked the migration and invasion induced by YB-1 overexpression (Figure 6B and 6C).

We explored the ability of inhibitors targeting STAT3 (S3I-201) and MEK (PD98059) to inhibit IL-6 signaling. These are key IL-6–regulated proteins that can also activate YB-1. Both STAT3 and MEK inhibition blocked IL-6 induction of morphological changes (Figure 6D) and the combination of the two inhibitors blocked IL-6 phosphorylation of YB-1 (Figure 6E). Critically, inhibition of either STAT3 or MEK was sufficient to maintain the epithelial morphology of MCF7 cells following IL-6 treatment.

### High levels of YB-1 and Stat3^S727^ correlate in both breast cancer cell lines and human breast tumors

**Figure 7:**
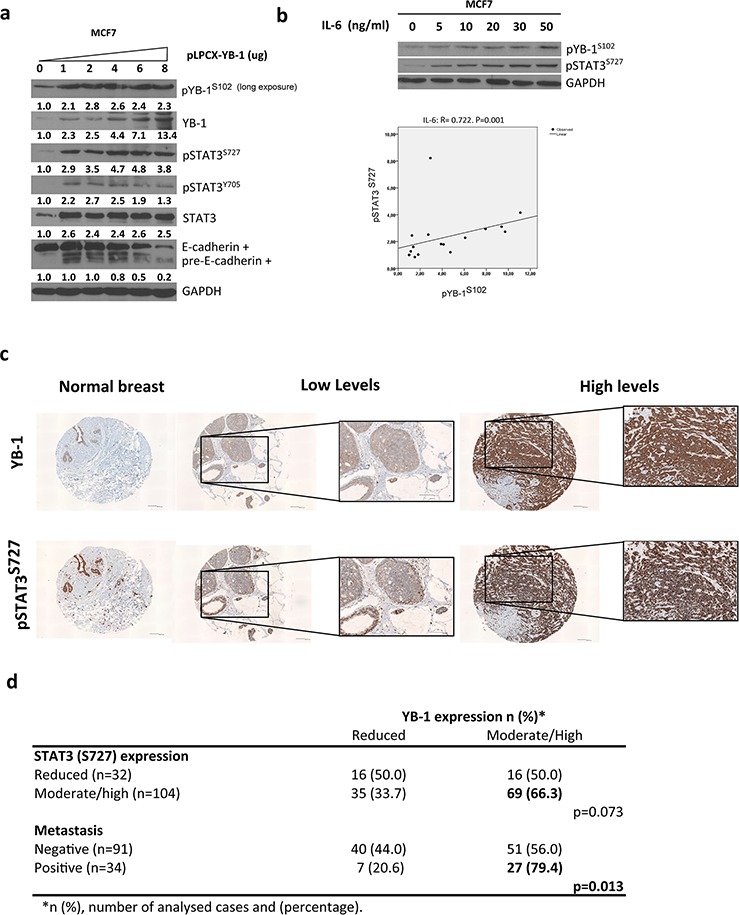
YB-1 and pSTAT3S727 are present in most human invasive/aggressive breast cancer types **a.** Western blot analysis of MCF7 cells transfected with increasing amounts of pLPCX-YB-1. **b.** Dose-dependent correlation between pYB-1^S102^ and pSTAT3^S727^ activation in breast cancer cells. Experiments were repeated twice, and bands were quantified using ImageJ software. Regression analysis was then conducted to determinate the correlation between pYB-1^S102^ and pSTAT3^S727^. **c.** Immunohistochemical staining for YB-1 and pSTAT3^S727^ in a tissue microarray of 138 high-grade breast carcinomas and normal breast samples. Shown is a representative sample of normal breast (left) and breast carcinoma samples with low and high expression of YB-1. Scale bar, 100 μm. **d.** Relationship between YB-1 amplification and clinicopathological features in high-grade breast cancer.

As discussed above, the IL-6 signaling pathway primarily involves activation of STAT3 by Tyr705 phosphorylation. However, STAT3 can also be phosphorylated at Ser727. The importance of Ser727 is still controversial because the pathways subsequently activated are different from those activated by Tyr705 [43]. In different cell models, the link between YB-1 and pSTAT3^S727^ affects cell survival [44, 45]. Therefore, we explored the levels of STAT3 phosphorylation at Ser727. Cells overexpressing YB-1 or IL-6 had increased levels of pSTAT3^S727^ (Figures 7A, S6A and S6B, S6C). Consistent with these results, high endogenous levels of YB-1 also correlated with elevated pSTAT3^S727^ levels in breast cancer cell lines (Figure S6D).

Next, we sought to analyze the correlation between YB-1 levels and metastasis in high-grade breast cancer tissues. Normal breast tissue expressed low levels of YB-1 and pSTAT3^S727^ (Figure 7C). In contrast, 62.5% of high-grade breast carcinomas presented moderate-to-elevated levels of YB-1 and 76.6% had elevated levels of pSTAT3^S727^ (Figure S6E). Of samples with elevated pSTAT3^S727^ levels, 66.3% were highly positive for YB-1 (Figure 7C and 7D). Of the tumors with metastasis, 79.4% exhibited moderate-to-high levels of positive staining for YB-1 (Figure 7D; *P* = 0.013). In high-grade breast tumors, YB-1 correlated with metastasis and tended to be associated with high levels of pSTAT3^S727^.

## DISCUSSION

Despite significant improvements in the diagnosis and treatment of breast cancer, recurrence and metastasis are still common and major causes of death. In the present study, we have provided new insights into the interaction between YB-1 and IL-6, two critical proteins in breast metastasis that can be pharmacologically targeted.

YB-1 is a powerful oncoprotein implicated in many cellular processes, including multidrug resistance [46] and metastasis [29]. Consistent with our data, YB-1 has been associated with aggressive types of breast cancer, which are mainly ER negative [10] and display a more invasive/metastatic phenotype. In agreement with this metastatic phenotype, overexpression of YB-1 induced EMT-like features in the breast cancer cells studied, such as increased cell migration and invasion. Forced YB-1 expression increased the levels of the mesenchymal marker *SNAI1* and downregulated *CDH1* (E-cadherin) mRNA levels. An inverse relationship between YB-1 and E-cadherin levels has been reported in many cancer cell lines and breast tumors [29, 32, 33, 47]. Upregulated levels of YB-1 can induce a cadherin switch, in which E-cadherin is replaced by N-cadherin [33, 48]. Nevertheless, it remains unclear whether YB-1 directly represses E-cadherin (transcriptionally or translationally) or acts indirectly by activating E-cadherin repressors such as Twist or Snail. In our setting, YB-1 knockdown in MDA-MB-231 cells reduced Snail1, Twist, and vimentin levels with a concomitant reduction in migration and invasion rates. However, we did not observe significant changes in E-cadherin at the protein level (data not shown) after YB-1 modification. Nevertheless, YB-1–depleted MDA-MB-231 cells adopted a more epithelial-like morphology, similar to other studies of MDA-MB-231 cells in which Snail1 was depleted [49] or E-cadherin was ectopically expressed [50–52]. Together, our results are consistent with prior findings [30–33, 53] showing that YB-1 promotes the acquisition and maintenance of an EMT-like phenotype.

IL-6 is a pleiotropic cytokine that modulates the phenotype of many cancer types by binding to IL-6 receptors and activating downstream pathways, thereby promoting tumor initiation, development, and metastasis [54]. We found that IL-6 increased pYB-1^S102^ in a similar manner to other growth factors such as EGF [30] or cytokines such as TGFβ or IL-2 [55]. Once YB-1 is phosphorylated, it mainly translocates to the nucleus where it promotes the transcription of genes involved in cell migration, invasion, drug resistance, and survival. Under stress conditions, YB-1 promotes cap-independent translation of pro-survival and growth genes [56], as well as transcriptional activation of drug resistance genes (*MDR1*) or drug transporters (*ABCC1* and *MVP1*). Our observations indicate that YB-1 may be involved in the survival of IL-6–or TGFβ-stimulated cells. Most of the cells depleted of YB-1 stop growing and die in the presence of one of these cytokines. We hypothesized that YB-1 could be involved in the signaling pathways of both cytokines and that its absence could be crucial for survival. One reason may be the positive association between YB-1 and the levels of activated STAT3, which is a pro-survival factor in several cancer cells. Interestingly, in an *in vivo* model of renal cancer, inhibition of YB-1 caused a concomitant decrease in STAT3 levels and increased sensitivity to cytokine IFN-α treatment. This combination had antitumor effects, indicating the pro-survival function of YB-1/STAT3 [45].

Our findings showed that YB-1 overexpression promoted the development of a metastatic phenotype that seems to be dependent upon IL-6 activity. It has been suggested that YB-1 can mediate part of these metastatic effects via the release of extracellular proteinases such as metalloproteinase 2 (MMP2), MMP13, MT1-MMP [29], and gelatinase A [57]. Interestingly, MMP release can be induced by IL-6 and some MMPs can regulate IL-6 expression. Hence, it is feasible that YB-1 would promote invasive effects through increased MMP secretion via IL-6 [58] or, alternatively, through a direct induction of MMPs that subsequently increase IL-6 expression [59]. YB-1 overexpression increased *IL6* mRNA and IL-6 secretion in the three cell lines tested. In view of these results and those of the RIP assay, it is feasible that YB-1 would be involved in the transcriptional regulation of IL-6, maybe as a factor located near the promoter region [60].

The idea that YB-1 is a transcription factor that activates IL-6 promoter activity is in agreement with a ChIP sequencing study in trastuzumab-resistant breast cancer cells that identified IL-6 and IL-6R as YB-1 transcriptional targets [61]. However, our YB-1/RNA co-immunoprecipitation assay indicated a direct binding with *IL6* at the RNA level, suggesting that YB-1 can be loaded on partially synthesized primary *IL6* RNAs to control aspects of RNA metabolism. Several studies have shown the ability of YB-1 to stabilize short-lived mRNA species such as IL-2 [62], GM-CSF [42], VEGF, and TGFβ [63], suggesting a link between YB-1 and post-transcriptional regulation of cytokines. Based on our RIP assay results, YB-1 can bind *IL6* RNA and may stabilize it, which is in accordance with a previous study [64] that showed that YB-1 regulates *IL6* mRNA stability in immune cells. Breast cancer cells overexpressing YB-1 had increased IL-6 production. This finding agrees with the evidence indicating that cancer cells secrete cytokines and chemokines to promote tumor progression via feed-forward loops in autocrine and paracrine manners. Autocrine loops are highly relevant in cancer progression and, in particular, the importance of IL-6 autocrine signaling in breast cancers is well known [18, 65]. In the present study, we found that IL-6 both induced and required YB-1 in order to activate EMT-like characteristics, including increased motility. On the other hand, the induction of an EMT phenotype by YB-1 required IL-6 signaling. Both proteins seem to create a positive feed-forward loop between each other that could explain the frequent co-expression of YB-1 and STAT3 [66].

IL-6 exerts most of its target gene expression functions via STAT3. The transcriptional activity of STAT3 is generally controlled by Tyr705 phosphorylation. Ser727 phosphorylation represents an additional level of regulation that increases STAT3 transcriptional activity [67], but the mechanism remains unclear. Although our results were not statistically significant, we found a tendency for an association between high levels of YB-1 and pSTAT3^S727^ in high-grade breast tumors. As pointed out in other tumor types, high levels of YB-1 positively correlate with metastasis [13, 29].

In conclusion, we have shown that (i) YB-1 overexpression promotes EMT features, cell migration, and invasion in breast cancer cell lines; (ii) YB-1 knockdown partially reverts the EMT phenotype of MDA-MB-231 cells; (iii) YB-1 regulates the IL-6/STAT3 pathway by inducing IL-6 *de novo* synthesis and activity; (iv) IL-6 is required for YB-1–induced acquisition of EMT-like features; (v) IL-6 can activate *YBX1* mRNA expression and YB-1 phosphorylation/activation; and (vi) induction of EMT by IL-6 requires YB-1. Elucidating the signaling pathways that govern the interdependence between IL-6 and YB-1 may uncover novel therapeutic approaches. These therapies might be particularly effective in patients with aggressive breast tumors that express high levels of YB-1 and pSTAT3.

## MATERIALS AND METHODS

### Cell culture

Breast cancer cell lines were purchased from the American Type Culture Collection (ATCC, Manassas, VA). MCF7 cells were maintained in RPMI 1640 medium while MDA-MB-231 and MDA-MB-468 cells were maintained in Dulbecco's modified Eagle's medium (DMEM) (Invitrogen, El Prat del Llobregat, Spain). Both media were supplemented with 10% heat-inactivated FBS (Invitrogen) and 1% antibiotic/antimycotic solution (10,000 U/mL penicillin G, 10 mg/mL streptomycin, and 25 μg/mL amphotericin B).

### Reagents and chemicals

Recombinant human IL-6 and EGF were purchased from Sigma-Aldrich (H7416 and E9644), reconstituted in phosphate-buffered saline, and used at final concentrations of 25 and 50 ng/mL, respectively. TGFβ was purchased from Sigma-Aldrich and used at a final concentration of 5 ng/mL. The MEK1/2 inhibitor PD98059 and STAT3 inhibitor S3I-201 were purchased from Calbiochem, dissolved in dimethyl sulfoxide (DMSO), and used at final concentrations of 50 μM and 100 μM, respectively. Cells were preincubated with the inhibitors for 1 h at 37°C and then incubated in the absence or presence of IL-6. The experiments ran for 4 days and media with inhibitor was changed every 2 days. BIX15 was purchased from Vibrant Pharma (Brantford, ON), dissolved in DMSO, and used at a final concentration of 5 μM. Control cells were treated with the same amount of DMSO (Vehicle).

### Modulation of expression using retroviral and lentiviral infection

Five micrograms of plasmids containing full-length cDNA of human *YBX1* in an pLPCX retroviral vector (pLPCX-YB1), cDNA of human *IL6* in a pLNCX (pLNCX-IL-6) vector, and an empty pLPCX retroviral vector (pLPCX) were used to generate viral supernatant in Phoenix packaging cells transfected with jetPEI^®^ (Polyplus, Illkirch, France) according to the manufacturer's protocol. Viral production and infection were performed at 37°C. All of these plasmids were sequenced twice from both sides to ensure expression of the correct coding sequence.

Regarding lentiviral shRNA, pLKO.1-puro-YB-1#1 and human *YBX1*#2 targeted 5′-CAGGCGAAGG TTCCCACCTT A-3′ and 5′-CAAGAAGGTC ATCGCAACGA A-3′, respectively. As a control, we used pLKO-shLUC, which targets human LUC. Production of lentiviruses and retroviruses and their infection of target cells were performed as previously described [68]. Infected MDA-MB-231 or MCF7 cells were selected with 2 μg/mL puromycin for 3–4 days; MDA-MB-468 cells were selected with 1 μg/mL puromycin for 3–4 days; and pLNCX-IL-6 cells were selected with 400 μg/mL G418 for 7 days.

### Knockdown analysis using siRNA transfection

Cells were reverse-transfected in 6-well plates with 10 nM siYB-1 or 50 nM siSTAT3 with RNAiMAX (Invitrogen) reagent according to the manufacturer's protocol. Double-stranded RNA purchased from Qiagen targeting *YBX1* (siYB-1#1: Hs_YBX1_1 FlexiTube siRNA SI03019191; siYB-1#2: Hs_YBX1_3 FlexiTube siRNA SI04172007), *STAT3* siSTAT3#1 (Dharmacon Research, Inc.), siSTAT3#2 (Qiagen, Hilden, Germany), or non-silencing control (FlexiTube Control siRNA SI03650325) were used. Cells were assessed after 72 h of siRNA transfection.

### Protein extraction and immunoblotting

#### Total cellular extraction

Total protein extracts were generated using RIPA buffer (50 mM Tris-HCl, pH 7.4, 150 mM NaCl, 1% Triton X-100, 1% sodium deoxycholate, 0.1% SDS, 1 mM EDTA) supplemented with PhosSTOP and Complete Phosphatase/Protease Inhibitor Cocktails (Roche Diagnostics GmbH, Mannheim, Germany). Protein extracts (20–25 μg per sample) were loaded onto gels and immunodetection of proteins was performed using ECL™ Western Blotting Detection Reagents (GE Healthcare, Buckinghamshire UK).

#### Extraction of cytoplasmic and nuclear proteins

Cytoplasmic and nuclear proteins extracts were prepared with NE-PER^®^ Nuclear and Cytoplasmic Extraction Reagents (Thermo Scientific, Rockford, IL) according to the manufacturer's description. The following primary antibodies were used: anti-YB1 (Millipore), 1:20000; anti-pYB1 S102 (Cell Signaling), 1:1000; anti-actin (Sigma, St. Louis, MO), 1:5000; anti-STAT3 (Cell Signaling), 1:1000; anti-STAT3 S727 (Cell Signaling), 1:1000; anti-STAT3Y705 (Cell Signaling), 1:1000; anti-Snail (Cell Signaling), 1:1000; anti-Twist (Santa Cruz Biotechnology), 1:1000; anti-vimentin (Santa Cruz Biotechnology), 1:1000; anti-GAPDH (Santa Cruz Biotechnology), 1:1000; anti-vinculin (Sigma), 1:1000; anti-LaminAC (Sigma), 1:10.000. Secondary antibodies (Calbiochem, La Jolla, CA) were used at 1:15000. Signals were scanned and quantified using ImageJ software.

### Quantitative real-time PCR

Total RNA was extracted from cells using RNAzol^®^ RT (MRC Inc., Cincinnati, OH) and 1 μg total RNA was used to synthesize cDNA using SuperScript^®^ III Reverse Transcriptase (Invitrogen, Life Technologies). qRT-PCR was performed on a Veriti 96-well Thermal Cycler (Applied Biosystems). TaqMan^®^ Gene Expression Assays were purchased from Invitrogen (*YBX1*: Hs02742755_g1; *CDH1*: Hs00170423_m1; *IL6*:Hs00985639_m1; *SNAI1*: Hs00195591_m1; and *ACTB*: Hs99999903_m1). To quantify changes in gene expression, the comparative *C*_t_ (ΔΔCT method) was used to calculate the relative fold change normalized to actin.

### RNA-binding protein immunoprecipitation

MDA-MB-231 cells were transfected with empty vector or pLPCX-YB-1 vector as described above. RNA-binding protein assay was performed with Magna RIP™ (Millipore, Billerica, MA) following the manufacturer's protocol. Mock control was done using a rabbit IgG and sample RNA-binding proteins were obtained using YB-1 IgG (Millipore). RNA-protein complexes were eluted at 95°C. Eluates were treated with proteinase K and RNA was ethanol-precipitated after extraction with phenol:chloroform:isoamyl. RNA quality was assessed using a NanoDrop. After treatment with DNaseI (Fermentas, Thermo Scientific), mRNA was subjected to cDNA synthesis and qRT-PCR was performed as described above. Fold enrichment was calculated as 2^−[(*C*t IP)–(*C*t mock)]^. This normalization is also referred to as “signal over background” or “relative to the no-antibody control”. With this method, the RNA-IP signals are divided by the no-antibody signals, representing the RNA-IP signal as the fold increase in signal relative to the background signal.

### IL-6 ELISA

ELISA was performed with an IL-6 ELISA kit (Invitrogen). Breast cancer cell lines with or without YB-1 were harvested at a concentration of 1.5 × 10^6^ in p60 plates, washed twice in medium (DMEM), and incubated in serum-free medium. Supernatant samples were harvested at 16 h and IL-6 levels were analyzed by following the manufacturer's instructions. Briefly, supernatant samples were added to wells in triplicate and incubated for 1 h at RT. They were then washed three times and incubated in anti–IL6-HRP conjugate solution for 1 h at RT, washed as described, and incubated with chromogen for 15 min at RT. The wells with the chromogen substrate were read at a wavelength of 490 nm with a microplate reader. Quantitative data are presented as average concentrations in pg/mL.

### Three-dimensional morphogenesis assay (semi-fluid basement membrane)

Growth factor-reduced Matrigel basement membrane (BD Biosciences, San Jose, CA) was added at a concentration of 40 μL per well to a 96-well plate and solidified by incubation at 37°C for 30 min. MCF7 cells stably transfected with empty vector (pLPCX) or YB-1–expressing vector (pLPCX-YB-1) in a single-cell suspension were added to the Matrigel wells in their respective media at 2 × 10^3^ cells/well. Colony growth was assessed in fields photographed 10 days after seeding.

### Cell migration (wound healing) assay

Cells were plated in 24-well plates in triplicate; 16 h before wounding of the confluent monolayer by scratching, the medium was removed and replaced by serum-free medium. Photomicrographs were taken and wound closure was measured using ImageJ software.

### Cell invasion assay

Invasion assays were carried out in modified Boyden chambers with Transwell inserts of 8-μm pore filters for 24-well plates (Millipore). Transwell inserts were coated with 70 μL diluted Matrigel basement membrane (BD Biosciences) for 30 min at 37°C. The cells were detached with trypsin and washed twice with serum-containing medium. Next, 200 μL of cells (2.5 × 10^5^ cells/mL) were resuspended and seeded in the upper chamber in serum-free medium in the presence or absence of IL-6 (20 ng/mL). Complete medium was placed in the lower compartment as a chemoattractant. Cells were also transfected with the appropriate siRNA for 48 h and then collected and resuspended in serum-free medium in the presence or absence of IL-6 (20 ng/mL). These cells were seeded in the upper chamber for 24 h at 37°C in a 5% CO_2_ humidified incubator. Non-invading cells on the top of the matrix were removed by rubbing the matrix with a moistened cotton swab. Cells that migrated through the permeable membrane were fixed in 3.7% formaldehyde, permeabilized with methanol, washed twice with phosphate-buffered saline, and stained with 0.2% crystal violet. Six random fields were photographed and cells were counted using ImageJ software.

### Immunohistochemical staining

A tissue microarray containing 138 high-grade breast tumor specimens was stained as described previously [69].

### Statistical analysis

Statistical analysis of the tissue microarray correlations between STAT3 phosphorylated at serine 727 (pSTAT3^S727^) and YB-1 staining in the clinical samples were determined using the Spearman's test. For our analysis, YB-1 was scored as 0 (negative) or 1(low levels), 2 (moderately positive), and 3 (highly positive). To determine the association between the presence/absence of YB-1 and the presence of pSTAT3^S727^, we used the chi-square test. Results were considered statistically significant at a *P* value of < 0.05.

Differences in continuous variables between groups were analyzed for statistical significance with one-way analysis of variance (ANOVA) followed by Tukey's post-hoc test using PASW 18 software (Chicago, IL). When the variance was not homogenous, we used the non-parametric Mann-Whitney *U* test or the Kruskal-Wallis test. Results are expressed as the mean ± SEM of at least three independent experiments and differences were considered significant at *P* < 0.05. In invasion assays, statistical analysis was performed by applying a two-sample *t* test assuming equal variance, and *P* values were calculated based on a two-tailed test.

## SUPPLEMENTARY FIGURES



## Abbreviations

BLBC: basal-like breast cancer
DMSO: dimethyl sulfoxide
EGF: epidermal growth factor
EMT: epithelial to mesenchymal transition
ER: estrogen receptor
IL-6: interleukin 6
MEK: mitogen-activated protein kinase kinase
MMP: metalloproteinase
PR: progesterone receptor
shRNA: short hairpin RNA
siRNA: short interfering RNA
TNBC: triple-negative breast cancer
YB-1: Y-box binding protein.
