# Supplementary material for: Interplay between YB-1 and IL-6 promotes the metastatic phenotype in breast cancer cells
Source: Oncotarget. 2015 Oct 15;6(35):38239–56. doi: 10.18632/oncotarget.5664 (PMC4741996; doi:10.18632/oncotarget.5664)
Supplement: Supplementary file 1 [file oncotarget-06-38239-s001.pdf]

SUPPLEMENTARY FIGURES

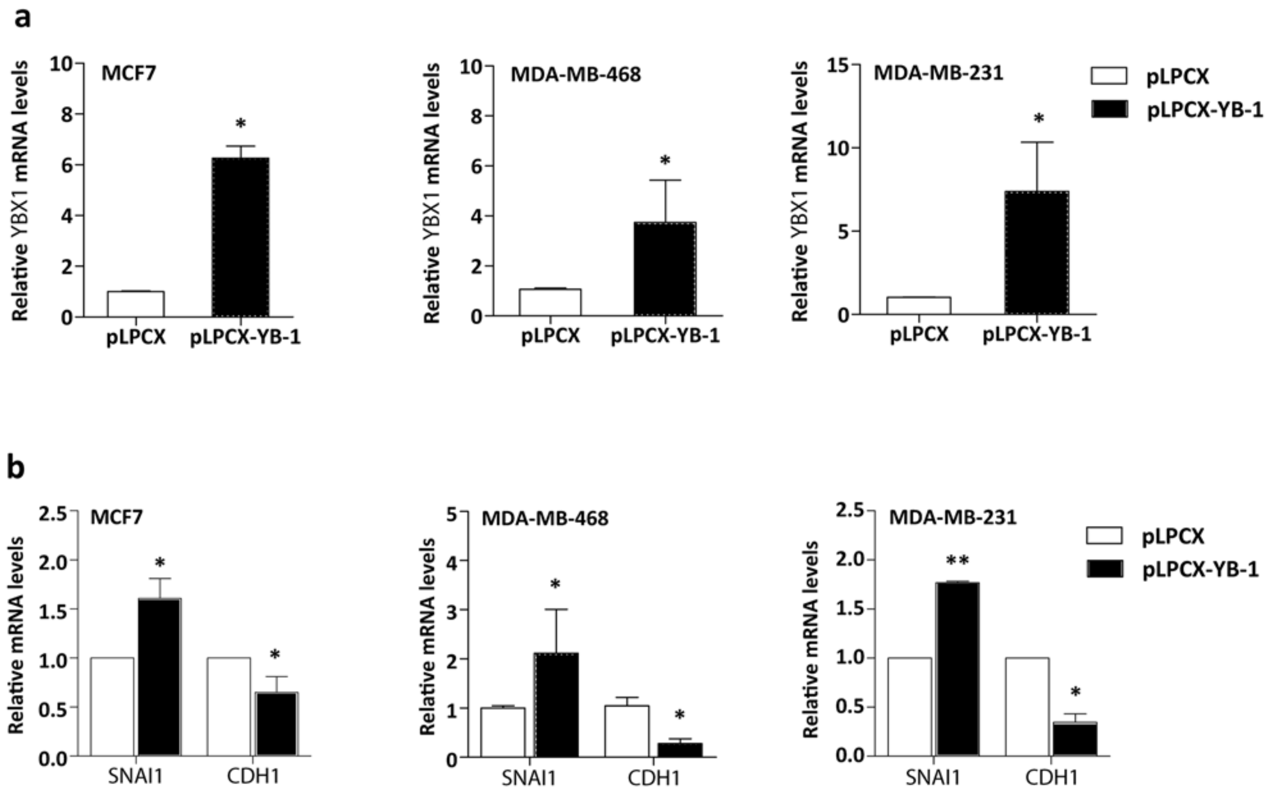

Supplementary Figure S1: YB-1 regulates CDH1 and SNAI1 expression in three breast cancer cell lines. qRT-PCR analysis of the mRNA levels of **a.** *YBX1*, **b.** the EMT marker *SNAI1*, and the epithelial marker *CDH1* (E-cadherin) in three breast cancer cell lines overexpressing YB-1 compared with control. The indicated means are significantly different to their controls. *$P < 0.05$, **$P < 0.01$ by the Mann-Whitney *U* test.

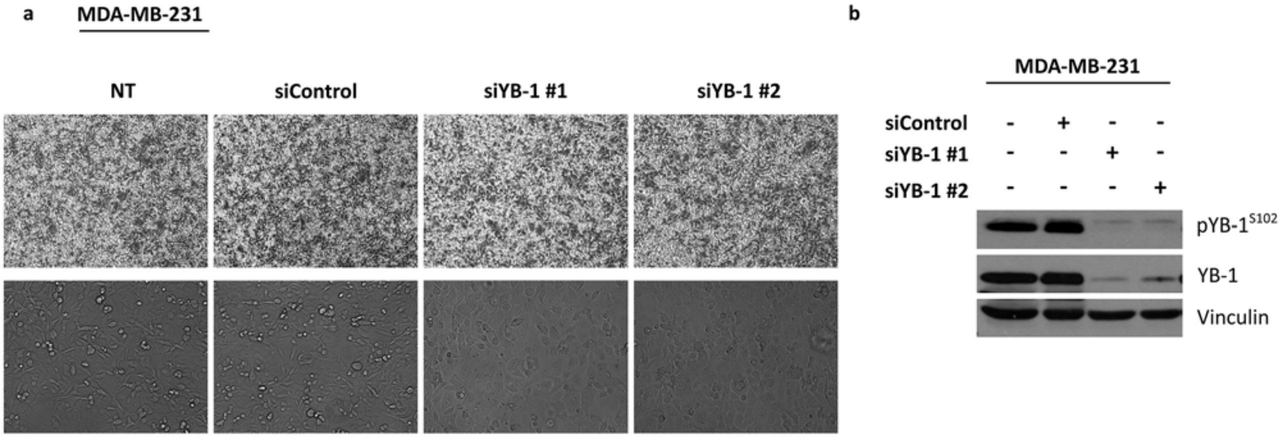

Supplementary Figure S2: Silencing YB-1 abrogates mesenchymal morphology in MDA-MB-231 cells. Cells were transfected twice with siRNAs targeting YB-1. The second transfection was performed 96 h after the first transfection and cells were incubated for 72 h more. **a.** Phase contrast microscopy images. **b.** Western blot analysis of pYB-1^{S102}, total YB-1, and actin.

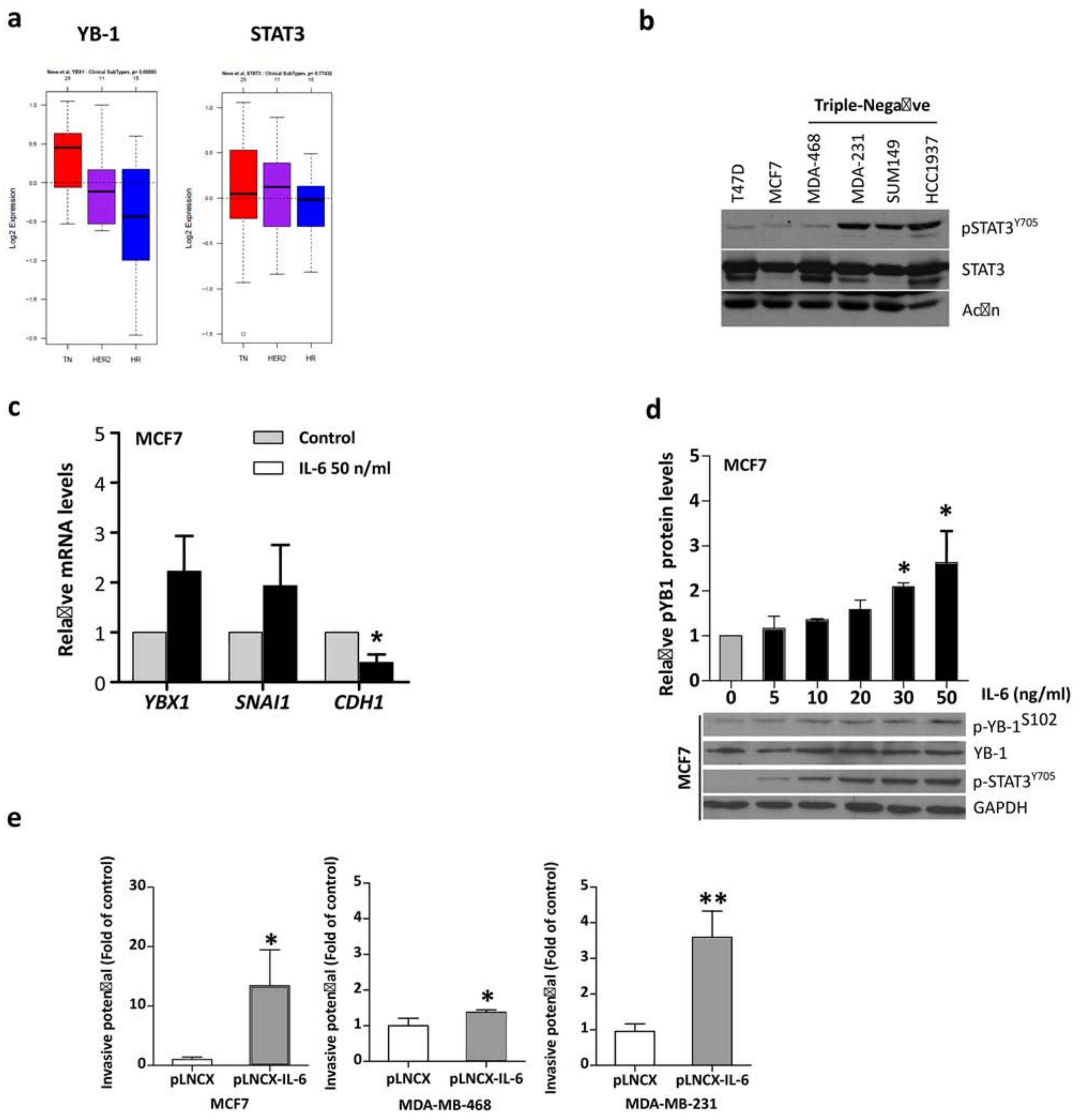

Supplementary Figure S3: IL-6 expression is associated with EMT characteristics. a. GOBO analysis of mRNA expression in breast cancer cell lines. **b.** Western blot showing the expression of $pSTAT3^{Y705}$, total STAT3, and actin in a panel of breast cancer cell lines. **c.** qRT-PCR analysis of *IL6*, *SNAI1*, and *CDH1* genes in MCF7 cells after IL-6 treatment. **d.** Dose–response correlation between $pSTAT3^{Y705}$ and YB-1 activation ($pYB\text{-}1^{S102}$). MCF7 cells were incubated in serum-free media for 16 h and treated for 4 h with IL-6 at the indicated concentrations. Whole cell extracts were subjected to Western blot. Band intensities of $pYB\text{-}1^{S102}$ and YB-1 were quantified and normalized to the intensity of GAPDH. Experiments were performed three times and data are presented as the mean ± SEM. $^{*}P < 0.05$ by Tukey's test. **e.** Cell invasion was assayed in MCF7, MDA-MB-468, and MDA-MB-231 cells stably expressing IL-6 and control vectors. Results are presented as the fold change over each parental cell line (mean ± SEM; $^{*}P < 0.05$, $^{**}P < 0.01$ by Student's *t* test). TN, triple-negative; HER2, HER2-overexpressing breast cancers; HR, hormone receptor-positive breast cancers.

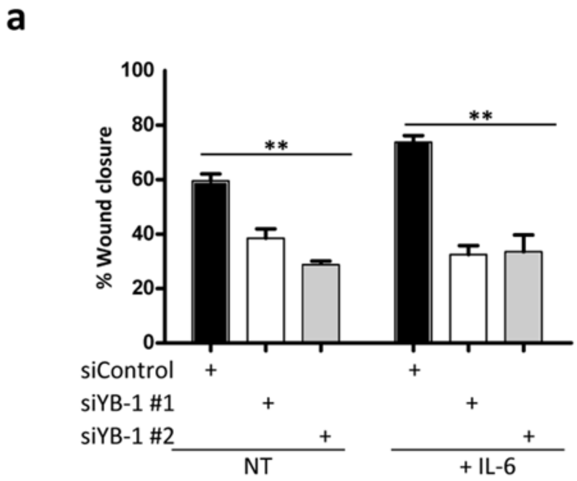

Supplementary Figure S4: Silencing of YB-1 reduces migration in MDA-MB-231 cells. a. Wound healing migration assay in siRNA-transfected cells (siYB-1 and siControl) in cells without or with IL-6 treatment. Cells were cultured in serum-free medium during the experiment. The indicated means are significantly different. *$P < 0.05$, **$P < 0.01$ by Tukey's test.

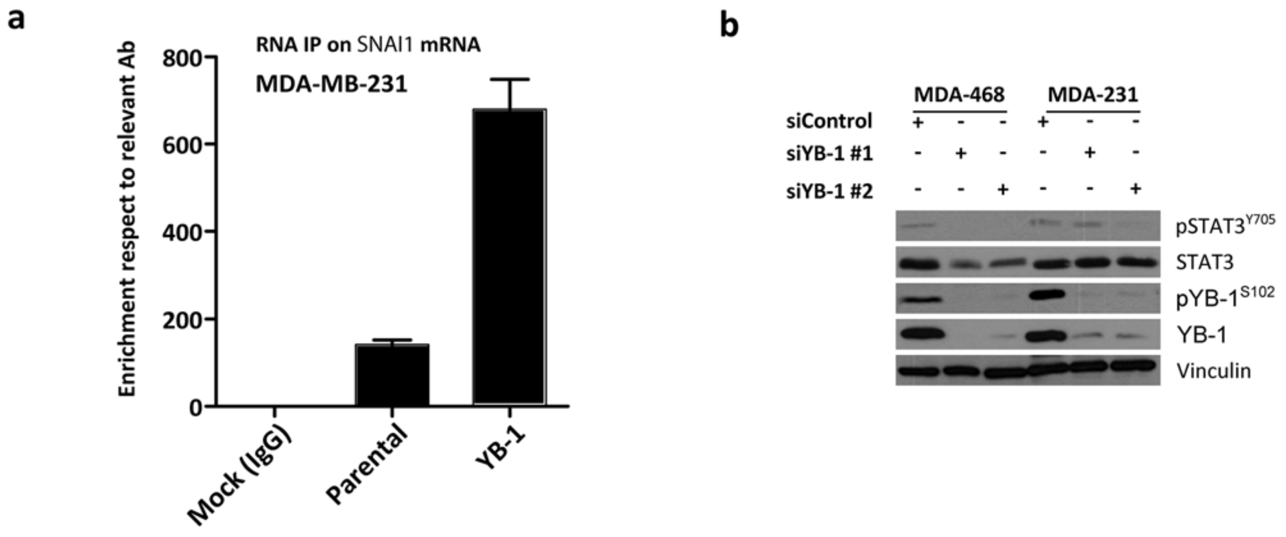

Supplementary Figure S5: Silencing of YB-1 reduces IL-6 expression. a. RIP assay of *SNAI1* in MDA-MB-231^{YB-1} cells compared with control cells. **b.** Western blot of MDA-MB-468 and MDA-MB-231 cells transfected with two YB-1–targeted siRNAs.

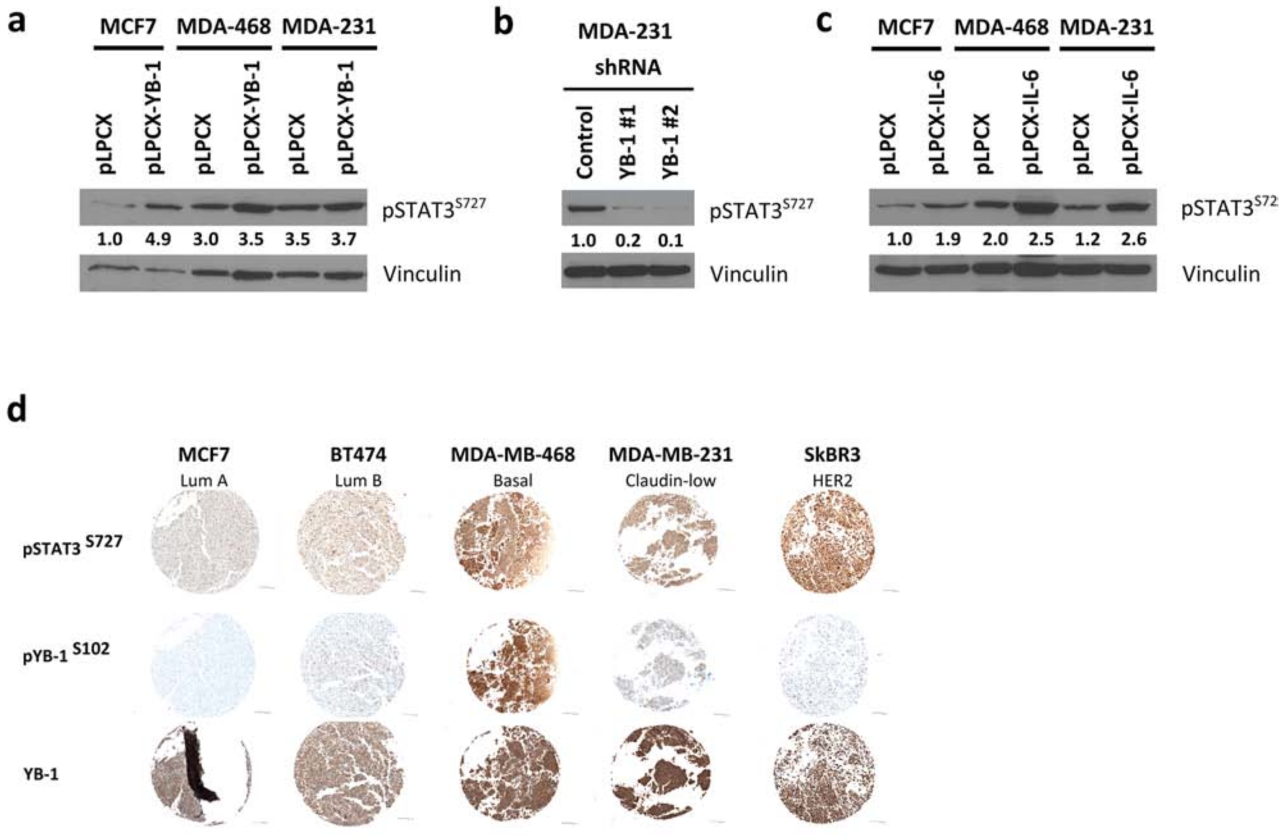

e

	n (%)
**YB-1** (n=138)	
Reduce	51 (37.5)
Moderate/High	85 (62.5)
**STAT3 (S727)** (n=137)	
Reduce	32 (23.4)
Moderate/High	105 (76.6)
**Metastasis** (n=127)	
No	92 (71.4)
Yes	35 (27.6)

Supplementary Figure S6: Expression of pSTAT3^{S727} is associated with YB-1 levels. Expression of pSTAT3^{S727} and endogenous protein was assessed by Western blot in **a.** YB-1–overexpressing cells, **b.** IL-6–overexpressing cells, and **c.** YB-1–depleted MDA-MB-231 cells. **d.** Immunohistochemistry of a breast cancer cell line tissue microarray. Levels of pSTAT3^{S727}, pYB-1^{S102}, and total YB-1 are shown. Scale bar, 100 µm. **e.** Summary of the clinicopathological and immunohistochemical features of the high-grade breast carcinoma tissue microarray.
